# Endobronchial Inflammatory Myofibroblastic Tumour Masquerading as a Ruptured Hydatid Cyst

**DOI:** 10.7759/cureus.58283

**Published:** 2024-04-15

**Authors:** Shiva S, Suresh Kumar, Pankaj Singh, Sanjeev Kumar, Vinita Agrawal

**Affiliations:** 1 Department of General Surgery, King George's Medical University, Lucknow, IND; 2 Department of Pathology, Sanjay Gandhi Postgraduate Institute of Medical Sciences (SGPGIMS), Lucknow, IND

**Keywords:** alk rearrangement, endobronchial tumor, complex hydatid cyst, pulmonary tumors, inflammatory myofibroblastic tumor

## Abstract

Inflammatory myofibroblastic tumours (IMTs) represent a rare group of neoplastic lesions characterized by a diverse clinical presentation. Endobronchial involvement is infrequently reported, and its manifestation mimicking the symptoms of a ruptured hydatid cyst adds an additional layer of complexity to the diagnostic challenge. This case report delves into an exceptional clinical scenario where an endobronchial IMT masqueraded as a ruptured hydatid cyst, initially confounding the diagnostic team. Through a detailed examination of the patient's clinical history, radiological imaging, bronchoscopy findings and subsequent histopathological analysis, we aim to contribute to the existing medical literature and shed light on the nuances encountered in accurately identifying and differentiating these two entities.

## Introduction

An inflammatory myofibroblastic tumour (IMT), also known as plasma cell granuloma, benign myofibroblastoma, lymphoid hamartoma, fibrous xanthoma pseudosarcoma myxoid hamartoma or inflammatory myofibrohistiocytic proliferation, is a histologically distinctive myofibroblastic spindle cell neoplasm of borderline malignancy commonly occurring in children and young adults and classically seen as an intermixture of plasma cells and lymphocytes with a prevalence rate of around 0.04-0.7% [1,2]. The usual sites of occurrence are the lung; abdominal cavity, particularly the greater omentum; mesentery; liver; and retroperitoneal space [3]. Endobronchial presentation is even rare, with an occurrence of around 12% of all reported cases [4]. Among the extra-pulmonary IMTs, around 11% are found to be in the upper respiratory tract, involving the larynx, trachea and oropharynx; 5% involve the orbits, paranasal sinuses, major salivary glands, thyroid and soft tissue [5]. They are usually asymptomatic, with only 20% of patients presenting with symptoms of generalised malaise, fever and weight loss [2]. Here, we present an exceptional clinical scenario where an endobronchial IMT masqueraded as a ruptured hydatid cyst, warranting the need for an urgent thoracotomy, implying the importance of considering uncommon differential diagnoses in pulmonary presentations that deviate from the expected norm.

## Case presentation

**Figure 1:**
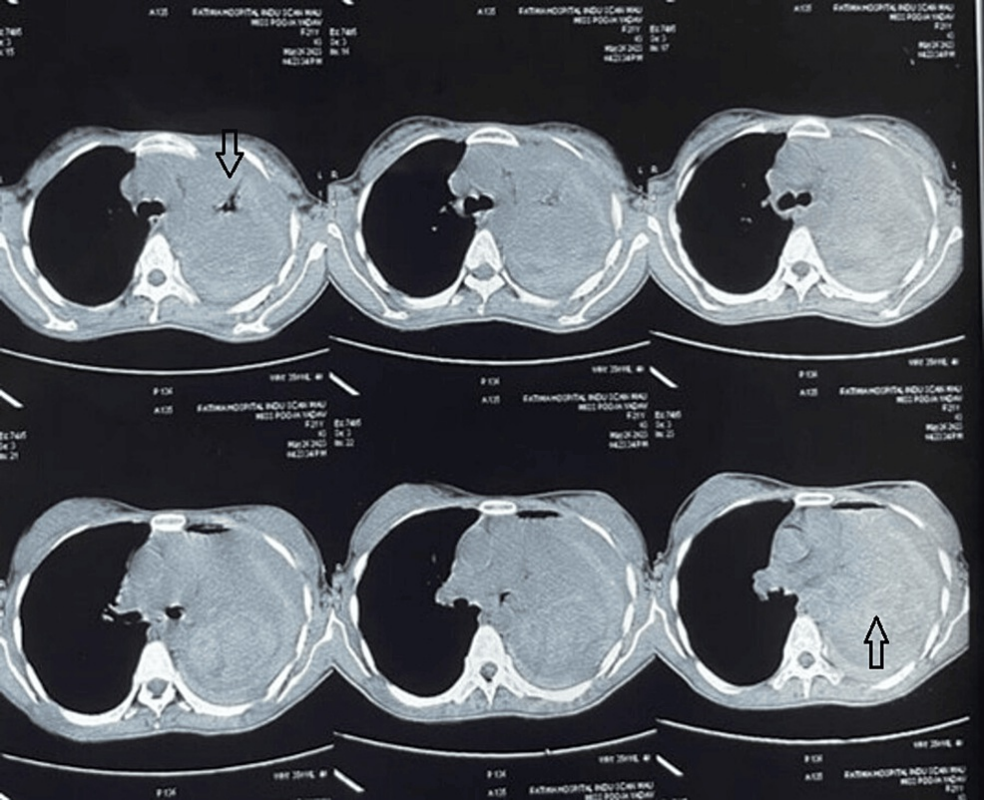
High-resolution computed tomography (HRCT) thorax showing left-sided pneumothorax with multiple membranes, thick septations, gross left-sided pleural effusion and collapse of the underlying lung parenchyma (arrow)

An unmarried, non-smoking female student in her 20s with no comorbid illness presented with complaints of left-sided chest pain and heaviness over the left side of her chest for six months. She started having symptoms six months ago, for which she had consulted outside. She was diagnosed with community-acquired pneumonia and was managed on an outpatient department (OPD) basis with antibiotics and analgesics. Her symptoms subsided completely. She developed cough, fever and chest pain again after six months and was admitted and treated with intravenous antibiotics. The tubercular workup was negative. A high-resolution computed tomography (HRCT) thorax was done, which showed left-sided pneumothorax with multiple membranes, thick septations, gross left-sided pleural effusion and collapse of the underlying lung parenchyma (Figure 1).

**Figure 2:**
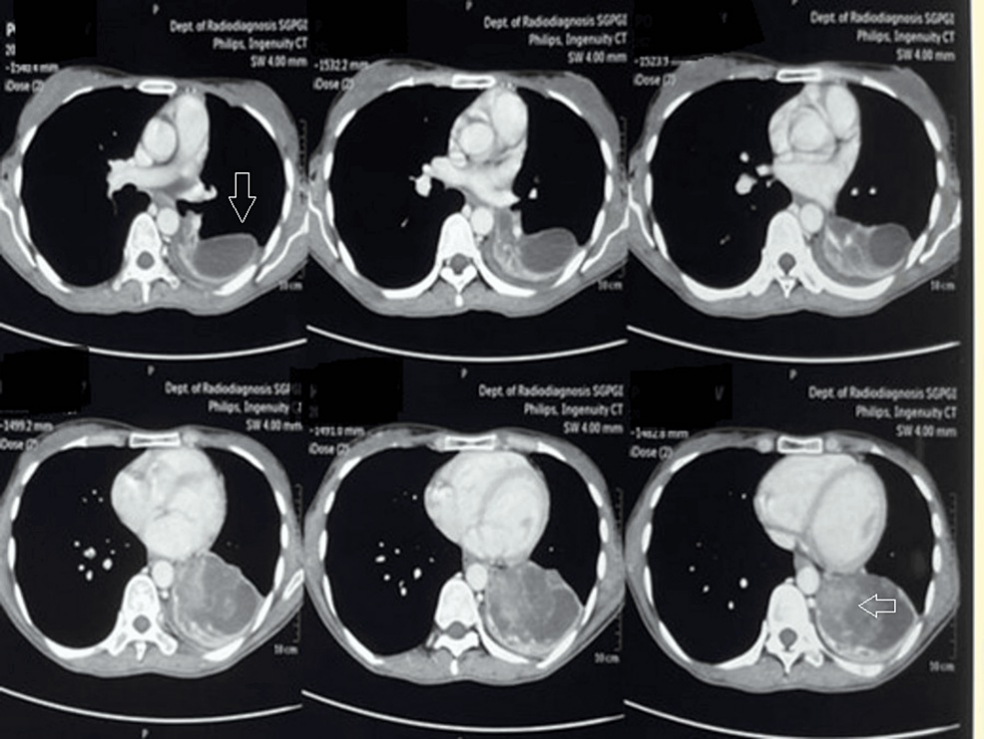
High-resolution computed tomography (HRCT) thorax showing an ill-defined heterogeneously enhancing lesion ~7.4 cm x 4.7 cm x 4.7 cm in the left lower lobe, infiltrating into the left lower lobe bronchus, with collapse of the left lower lobe (arrow)

Based on the HRCT findings, hydatid disease of the lung was suspected. *Echinococcus* enzyme-linked immunosorbent assay (ELISA) IgG was negative, and she was started on the anti-helminthic drug albendazole 400 mg twice daily. Her symptoms were alleviated but recurred after 20 days when she was referred to a higher centre. A repeat HRCT thorax was done, which showed an ill-defined heterogeneously enhancing lesion ~7.4 cm x 4.7 cm x 4.7 cm in the left lower lobe, infiltrating into the left lower lobe bronchus, with a collapse of the left lower lobe (Figure 2).

**Figure 3:**
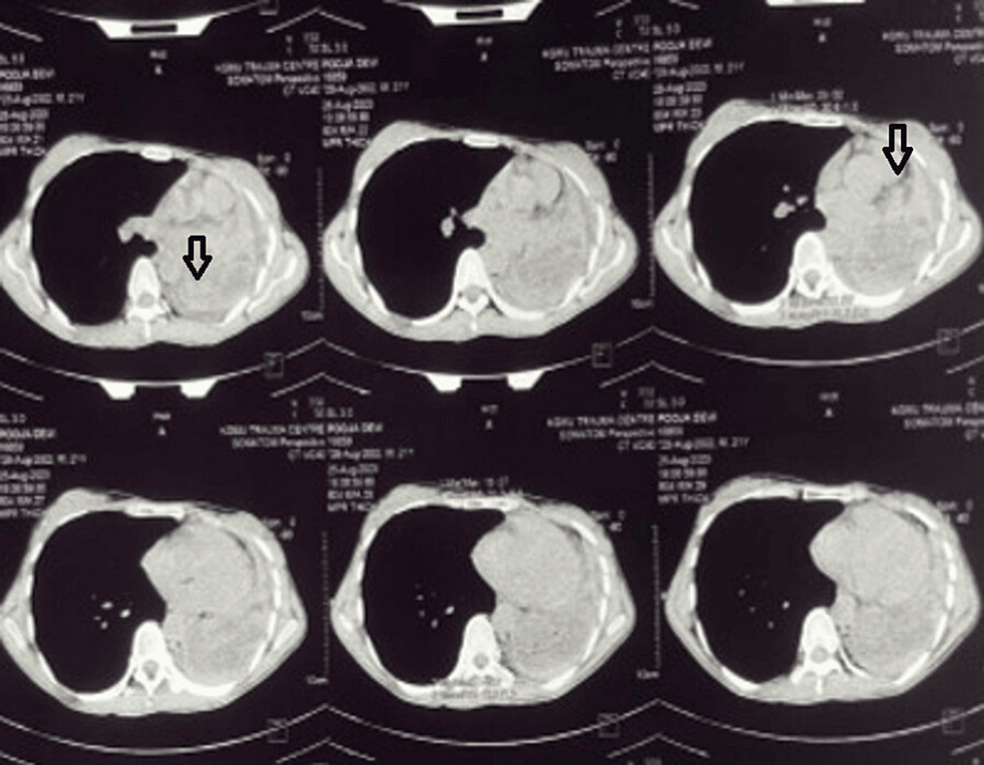
High-resolution computed tomography (HRCT) thorax showing left-sided space-occupying lesion infiltrating into the left main bronchus with collapse of the underlying lung parenchyma (arrow)

A repeat ELISA IgG for *Echinococcus* was done and found to be positive. The patient was again started on tab albendazole 400 mg twice daily, and there was alleviation of symptoms. After the initial response to anthelminthic treatment, symptoms recurred, and a bronchoscopy was done, which was suggestive of a cystic lesion of the left main bronchus, suspicious of a complex hydatid cyst, and referred to our centre for urgent surgical intervention. On presentation, the patient was tachypnoeic, requiring low-flow oxygen. Her respiratory system examination revealed inspiratory crackles in the left mammary region. Her blood counts, renal function tests, liver function tests, arterial blood gas analysis, erythrocyte sedimentation rate (ESR), C-reactive protein (CRP), chest X-ray and electrocardiogram were normal. A repeat HRCT was done, which was suggestive of a left-sided space-occupying lesion infiltrating into the left main bronchus with a collapse of the underlying lung parenchyma (Figure 3).

**Figure 4:**
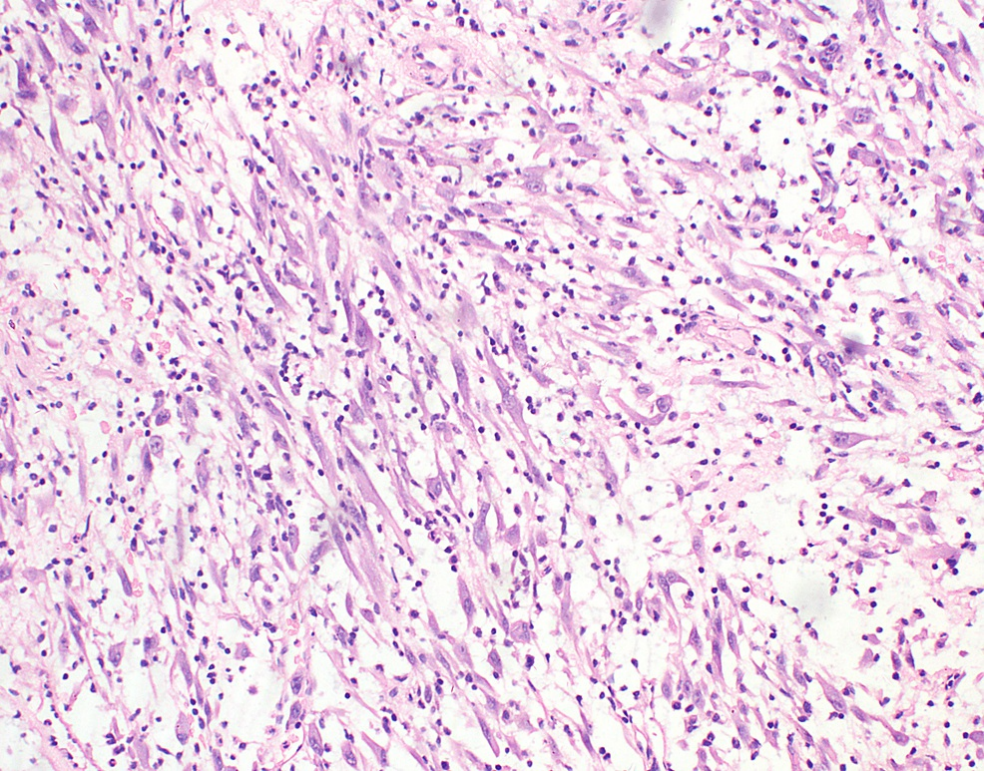
Histopathological examination of the mass showing a lesion composed of loosely arranged spindle cells in a myxomatous stroma, showing lymphocytes and plasma cells

In view of the high suspicion of a ruptured hydatid cyst, the patient was taken up for emergency thoracotomy via the left posterolateral approach. Intraoperatively, a mass arising from the left lower lobe involving the adjacent left main bronchus was seen. A biopsy was taken from the lesion, and intercostal chest tube drainage was placed. Histopathological examination of the mass showed a lesion composed of loosely arranged spindle cells in a myxomatous stroma, showing lymphocytes and plasma cells (Figure 4).

**Figure 5:**
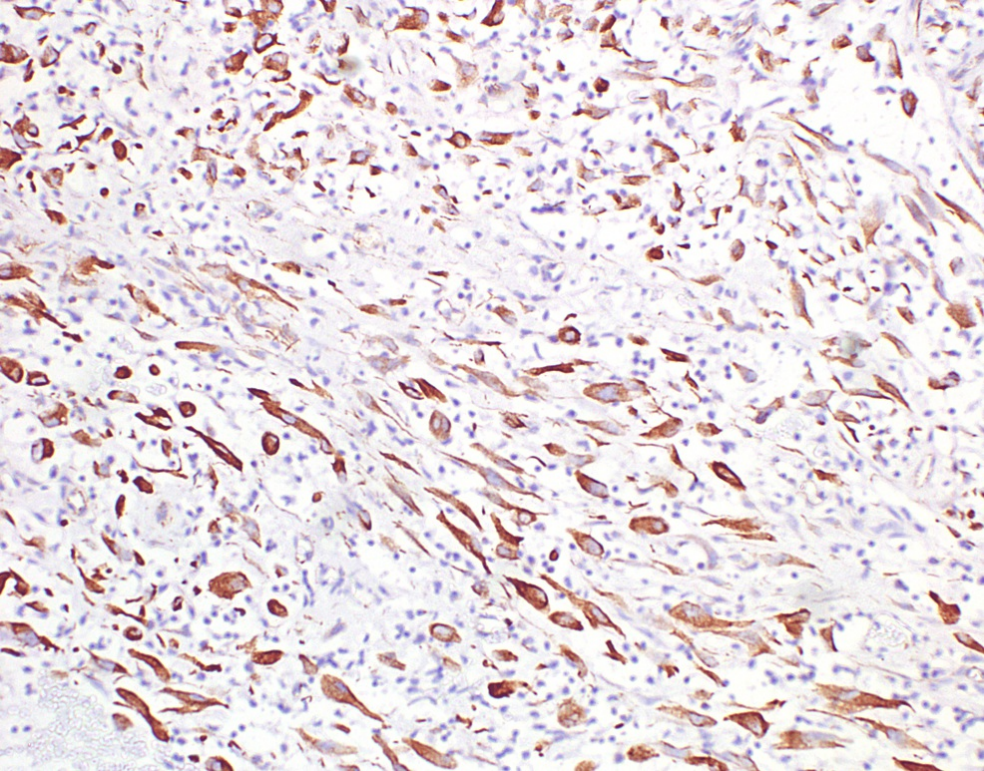
Immunohistochemistry of the spindle cells showing diffuse and strong expression for vimentin

**Figure 6:**
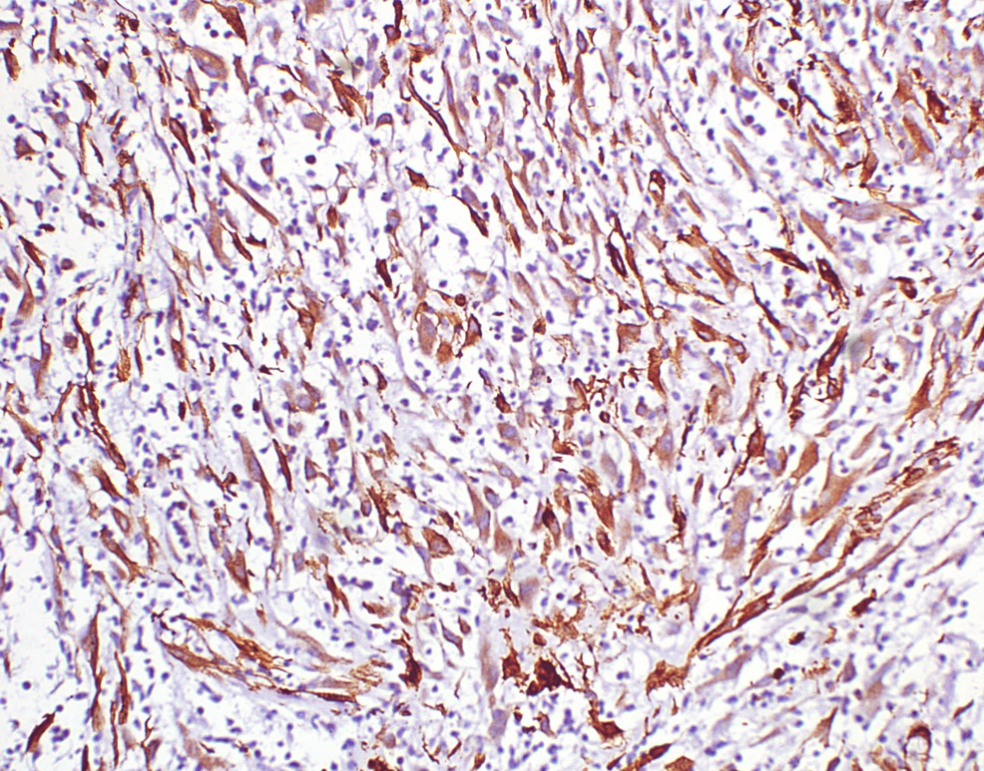
Immunohistochemistry of the spindle cells showing diffuse and strong expression for SMA

**Figure 7:**
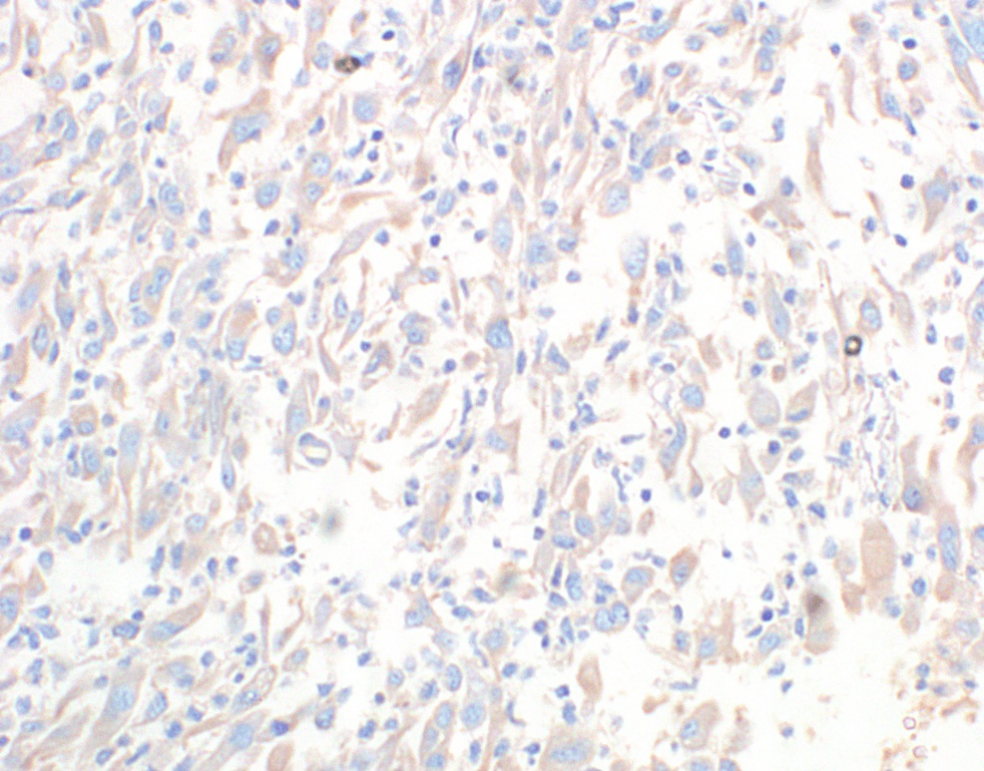
Immunohistochemistry of the spindle cells showing weak expression of ALK

The spindle cells display round to oval nuclei, dispersed chromatin, conspicuous nucleoli and a moderate amount of dense eosinophilic cytoplasm. There was no significant mitosis. Immunohistochemistry of the spindle cells showed diffuse and strong expression for vimentin (Figure 5) and SMA (smooth muscle actin) (Figure 6) and weak expression of anaplastic lymphoma kinase (ALK) (Figure 7); myogenin, desmin, cytokeratin, CD34 and STAT6 were negative.

The Ki67 proliferation index was <1%, suggestive of an IMT. The metastatic workup was negative. The patient was referred to a higher centre due to a lack of modalities for definitive treatment.

## Discussion

An IMT is a neoplasm composed of myofibroblastic and fibroblastic spindle cells with inflammatory cells like lymphocytes and eosinophils. IMTs most commonly arise from the lung, but the endobronchial presentation is rare, with a prevalence of 0-12% [6]. The incidence of pulmonary IMT in adults is around 0.04-1% of all lung tumours, with most tumours occurring in patients less than 40 years of age and without any gender predilection [6]. They are usually asymptomatic; if symptomatic, the signs and symptoms are associated with the tumour mass effect, swelling and local inflammation and vary depending on the anatomical location. Rare presentations like paraneoplastic pemphigus have also been reported [7]. The aetiology and pathogenesis of IMT are not well understood, but smoking, minor trauma, IgG4-related disease, human herpes virus 8, Ebstein bar virus, actinomyces, *Nocardia* and mycoplasma are the known risk factors [2,4].

Histologically, they show myofibroblastic spindle cell proliferation with mixed inflammation (lymphocytes, plasma cells and eosinophils) and a variable number of mitotic figures without significant pleomorphism. Various histological patterns, like loosely arranged myxoid or hyaline stroma, spindle to stellate cells and admixed inflammatory cells (nodular fasciitis-like), storiform or fascicular growing elongated spindle cells without overt hyperchromasia, cytologic atypia, associated with prominent lymphoplasmacytic infiltrate and hypocellular, scar-like pattern with occasional metaplastic bone or associated calcifications may be seen. Epithelioid variants are predominantly composed of plump round epithelioid cells with vesicular chromatin, large, prominent nucleoli, amphiphilic to eosinophilic cytoplasm, a prominent neutrophilic component and abundant myxoid stroma [8]. The histological differential diagnoses include nodular fasciitis, desmoid type fibromatosis, inflammatory leiomyosarcoma, IgG4-related sclerosing disease, Hodgkin's lymphoma, inflammatory fibroid polyp and gastrointestinal stromal tumour. Immunohistochemistry shows diffuse and strong vimentin positivity with variable smooth muscle actin, muscle-specific actin, calponin and desmin. Keratin is positive in 40-70% of the cases and ALK1 in 40-60% of the cases [9]. ALK-negative cases can be diagnosed by molecular studies if clinical suspicion is high. The epithelioid variant has a distinct nuclear membrane or perinuclear pattern of ALK staining [8,10]. Molecular characterization of IMTs shows ALK-4, PDGFRβ, ROS1 and RET rearrangements in 50-70% of the cases [11]. These genetic alterations result in the activation of the tyrosine kinase receptor and may offer novel possibilities for treatment with receptor kinase inhibitors in unresectable and metastatic cases.

The radiological presentation of lung IMTs is non-specific and usually presents as peripheral lung masses with a variable degree of contrast enhancement, which is very similar to malignant lung masses and often requires a biopsy for a definitive diagnosis [12]. The lesions can present radiologically as multiple nodular (5%), pleural-based, cavitary (5%), lobar atelectasis (8%) or hilar lymphadenopathy (5%) [13]. Positron emission tomography or CT is highly sensitive but has low specificity for IMT and could be useful to evaluate the response to treatment in patients not eligible for surgery.

Another point to ponder over is the positive IgG ELISA for *Echinococcus* in our case and its utility in the diagnosis of hydatid disease. A literature review shows low sensitivity (30% false negativity) and low specificity (25% false positivity), suggesting only a complementary role in the diagnosis of hydatid disease [14].

Due to the rarity of the disease, definite treatment guidelines are lacking, but surgical resection is a modality of treatment with a rarely reported recurrence after radical resection. Surgical resection can be done via video-assisted thoracoscopy or open thoracotomy. Wedge resection should be considered the first-line treatment, but lobectomy or pneumonectomy can be performed to ensure radical resection. Enbloc resection may be needed in cases of chest wall invasion, carina, main bronchus, pericardium or diaphragm involvement. Bronchoscopic resection has also been tried with success [15]. A five-year survival rate of 90% has been reported after complete resection of tumours less than 3 cm [4]. Local recurrence rates of 15-37% and metastasis rates of 5-11% have been reported [6]. Treatment with the anti-TNF-alpha antibody Infliximab has been tried with success [16]. Tao et al. have been successful in treating a retroperitoneal IMT with non-steroidal anti-inflammatory drugs (NSAIDs) and platinum-based chemotherapy [17]. The treatment with NSAIDs is based on cyclooxygenase-2 (COX-2) and VEGF inhibitory properties and can possibly explain the improvement of symptoms initially in our case. Khalil et al. reported complete remission with ALK inhibitor crizotinib, but because of reports of poor initial response or development of resistance after a few months, second-generation ALK inhibitors (ceritinib and alectinib) have been developed, which have shown promising results [5,18]. Argon plasma and cryotherapy have also been used as treatment modalities [19].

To conclude, this case report serves as a reminder of the complexities involved in the diagnosis of IMFTs and the necessity of a meticulous diagnostic approach. Further research and larger case series are warranted to better understand the diverse presentations of IMFTs and improve diagnostic accuracy, ultimately contributing to more effective and timely management of these rare tumours.

## Conclusions

This case highlights the importance of considering uncommon presentations of common diseases. In this instance, the presentation of IMFT masquerading as a ruptured hydatid cyst underscores the necessity for thorough diagnostic evaluation and clinical suspicion. Early recognition and appropriate management are crucial for optimal patient outcomes. This case serves as a reminder for clinicians to maintain a broad differential diagnosis and to utilize imaging and histopathological findings to guide accurate diagnosis and treatment strategies. It also highlights the complexities involved in the diagnosis of IMFTs and the necessity of a meticulous diagnostic approach. Further research and larger case series are warranted to better understand the diverse presentations of IMFTs and improve diagnostic accuracy, ultimately contributing to more effective and timely management of these rare tumours.
